# Decoding the Oncogenic Role of GNG10 in Colorectal Cancer: A Non‐Canonical Wnt Pathway–Driven Mechanism

**DOI:** 10.1111/jcmm.71170

**Published:** 2026-06-07

**Authors:** Xitao Zhang, Yuting Tang, Xuexiao Li, Ou Li, Yaoqian Liu, Jianping He, Tianlai Liu

**Affiliations:** ^1^ Department of Coloproctology Zhujiang Hospital of Southern Medical University Guangzhou China; ^2^ Department of Outpatient Sushe Street Community Health Service Center Guangzhou China

**Keywords:** cancer stemness, colorectal cancer, GNG10, non‐canonical Wnt pathway, tumour progression

## Abstract

Guanine nucleotide‐binding protein gamma 10 (GNG10) is implicated in various biological processes, yet its specific oncogenic role in colorectal cancer (CRC) remains poorly defined. This study aimed to elucidate the expression patterns, biological functions, and underlying mechanisms of GNG10 in CRC progression. We integrated TCGA datasets with tissue microarray immunohistochemistry and multivariate Cox regression models to evaluate the clinical significance of GNG10. Functional impacts on CRC malignant phenotypes and cancer stemness were assessed through gain‐ and loss‐of‐function models in vitro and in vivo. Mechanistic insights were gained via GSEA, Western blotting, and dual rescue strategies employing both pharmacological inhibition (Box5) and genetic depletion (shRHOA). We found that GNG10 was markedly overexpressed in CRC tissues, correlating with advanced pathological stage and poor overall survival. Multivariate analysis indicated that the prognostic value of GNG10 is closely associated with tumour progression. Functionally, GNG10 knockdown inhibited CRC cell proliferation, migration, and stemness while promoting apoptosis. Mechanistically, GNG10 activated the non‐canonical Wnt/RHOA/JNK/NFATc1 signalling axis. Crucially, manipulation of GNG10 did not affect active or total β‐catenin levels, thereby excluding canonical Wnt involvement. Both pharmacological inhibition with Box5 and genetic ablation of RHOA effectively abrogated GNG10‐induced oncogenic phenotypes and the upregulation of cancer stem cell markers (CD44, CD133, OCT4, Nanog, SOX2). In vivo xenograft models confirmed that GNG10 knockdown suppressed tumour growth and decreased the expression of proliferation and stemness markers. Our findings demonstrate that GNG10 promotes CRC progression and stemness via the non‐canonical Wnt signalling pathway. These findings highlight GNG10 as a promising prognostic indicator and a vulnerable target in CRC.

## 1 Introduction

As the third most frequent cancer and the second primary driver of cancer‐related deaths worldwide, colorectal cancer (CRC) represents a gastrointestinal malignancy characterized by a consistently rising occurrence and fatality rate in recent years. Based on international oncology data, CRC comprises nearly 10% of total cancer diagnoses and is the second most common origin of cancer fatalities, presenting a substantial strain on public health systems [1, 2, 3]. Established contributors to CRC include hereditary susceptibility, notably mutations within the APC and KRAS genes, alongside lifestyle influences like adiposity, physical inactivity, and a nutritional pattern rich in processed meats but deficient in fibre [4, 5, 6]. Notwithstanding progress in therapeutic approaches, encompassing surgery, chemotherapy, radiotherapy, and targeted therapies, the clinical outlook for CRC patients continues to be unsatisfactory, especially for those presenting advanced or metastatic stages. Existing therapies frequently encounter constraints, including pharmacological resistance, adverse toxicities, and insufficient effectiveness in enhancing overall survival [7, 8, 9]. These hurdles highlight the imperative to decipher the fundamental molecular pathways driving tumour advancement [10]. A comprehensive insight into these processes is essential for uncovering innovative therapeutic markers and designing superior clinical protocols, which may eventually enhance patient recovery and alleviate the worldwide impact of CRC.

Encoded by the GNG10 gene situated at the 9p13.3 chromosomal locus, Guanine nucleotide‐binding protein subunit gamma‐10 (GNG10) serves as a vital member of the guanine nucleotide‐binding protein (G protein) gamma subunit group and is widely distributed across diverse physiological sites, such as the cerebral, hepatic, and blood‐forming systems [11]. GNG10 functions as a pivotal regulator within G protein‐coupled receptor (GPCR) transduction networks, orchestrating internal signalling streams like the cAMP/PKA and MAPK cascades, which are fundamental for governing cellular activities including growth, maturation, and programmed cell death [11, 12, 13]. GNG10 has been increasingly recognized as a prospective pro‐tumourigenic factor within various malignancies. For instance, in glioma, GNG10 has been documented as a predictive indicator that may cross‐talk with the PI3K‐Akt signalling axis to modulate neoplastic development [12]. Similarly, aberrant expression of GNG10 has been linked to radiation sensitivity and clinical outcomes in head and neck squamous cell carcinoma (HNSCC) [14]. Notably, in CRC, GNG10 has been demonstrated to facilitate malignancy advancement via its engagement with the lncRNA CCAT1/miR‐4679 regulatory network, underscoring its role in tumour promotion [11].

Crucially, while GNG10 is recognized as a general oncogenic factor, its precise downstream signalling network in CRC remains poorly defined. Intriguingly, emerging evidence highlights a profound functional crosstalk between GPCR signalling and the Wnt cascade, a fundamental driver of CRC pathogenesis [15]. Wnt receptors, particularly the Frizzled (FZD) family, structurally resemble GPCRs and can directly activate heterotrimeric G proteins upon ligand binding [16]. Following this triggering, released Gβγ complexes—where GNG10 serves as a central component—chiefly propagate signals via β‐catenin‐independent, non‐canonical Wnt routes, including Wnt/planar cell polarity (PCP) and Wnt/Ca2+ signalling streams [17]. These non‐canonical axes are notorious for regulating cytoskeletal dynamics, cell migration, and cancer stemness via downstream effectors including RHOA and JNK [18]. In spite of these persuasive theoretical connections, the exact molecular processes through which GNG10 drives CRC advancement remain largely elusive, and its specific role in governing CRC progression via non‐canonical Wnt signalling remains largely uninvestigated [11]. In light of sparse data regarding the participation of GNG10 in tumour development, especially within CRC, this research was designed to clarify the functional importance of GNG10 in this malignancy and its feasibility as a clinical target. By filling this information void, our investigation intends to offer fresh perspectives on the molecular landscape of CRC and aid in the formulation of precision therapy approaches.

In this study, our main goal is to clarify the clinical relevance and oncogenic role of GNG10 in CRC progression, specifically focusing on its mechanistic connection to the non‐canonical Wnt pathway. We utilized an integrated translational framework to accomplish this. Initially, we integrated clinical bioinformatics from the TCGA database with rigorous tissue microarray validation to establish GNG10 as a robust prognostic indicator in CRC. Subsequently, by performing diverse laboratory‐based experiments and murine xenograft studies, we comprehensively evaluated the impact of GNG10 on tumour growth, malignant phenotypes, and cancer stemness. Crucially, combining pharmacological inhibition and genetic rescue strategies, we sought to delineate the precise downstream signalling cascade orchestrated by GNG10. Ultimately, our investigation seeks to reveal the essential function of the GNG10/RHOA/non‐canonical Wnt signalling module in promoting CRC progression, thereby establishing a compelling rationale for its feasibility as a fresh therapeutic target.

## 2 Materials and Methods

### 2.1 Bioinformatics Analysis

Computational investigations were conducted through The Cancer Genome Atlas (TCGA) repository. Transcriptomic profiling of GNG10: Comparing the levels of GNG10 in CRC malignant samples against adjacent non‐tumour counterparts involved RNA sequencing information retrieved via the Genomic Data Commons (GDC) platform (https://portal.gdc.cancer.gov/). Survival assessment: The correlation between GNG10 abundance and patient clinical outcomes was assessed based on follow‐up records from the cBioPortal for Cancer Genomics (https://www.cbioportal.org/). Pathway enrichment: GSEA was performed on RNA sequencing profiles within the TCGA‐COAD+READ cohorts (GDC resource, The Cancer Genome Atlas). Enrichment scores were calculated using GSEA_Linux_4.2.3, referencing gene collections from the Molecular Signatures Database (MSigDB), incorporating canonical pathways and Gene Ontology (GO) libraries. Transcriptome counts underwent normalization using the DESeq2 R software, and cohorts were stratified into high and low GNG10 categories based on their median values. Standard settings were applied, with the exception of 1000 permutations and gene set size filters (15–500 genes per cluster).

### 2.2 Clinical Sample Collection and Immunohistochemical Staining

Tissue microarrays (TMAs) representing CRC and normal colon segments provided from Shanghai Outdo Biotech Co. Ltd. (#HColA180Su15‐M‐102) were used. Ethical clearance for these samples was granted by the Medical Ethics Committee of Zhujiang Hospital, Southern Medical University, with informed consent secured from every participant.

Standard IHC procedures were strictly followed. Initially, slides were heated at 65°C for 30 min, cleared in xylene, and hydrated using descending ethanol concentrations (100%, 100%, 95%, and 75%) before rinsing. Epitope retrieval occurred in EDTA solution (pH 8.0) at 100°C for 30 min using a pressurized vessel. Endogenous peroxidases were quenched with 3% hydrogen peroxide (H_2_O_2_) for 10 min at ambient temperature. After rinsing with PBS, slides were treated with anti‐GNG10 primary antibody (Affinity, #DF2205, 1:200) overnight at 4°C. After rinsing, HRP‐conjugated secondary antibody was applied for 1 h at ambient temperature. Visualization was achieved via DAB, followed by haematoxylin counterstaining for 30 s. After drying, samples were fixed using neutral resin and coverslipped. Staining outcomes were scored semi‐quantitatively based on depth and coverage. Multiplying intensity (0–3) by positivity percentage (0–4), composite values were grouped into negative (0), weakly positive (1–4), moderately positive (5–8), and strongly positive (9–12). Two senior pathologists blindly evaluated the slides to guarantee diagnostic reliability.

### 2.3 Cell Culture and Reagent

The normal human colonic epithelial cell line FHC and five CRC cell lines (HT29, RKO, DLD‐1, HCT116, and CACO2) were employed for this research. Following preliminary baseline expression analysis, HCT116 and RKO were then selected for functional studies. FHC cells were grown in DMEM/F‐12 medium (Gibco) enriched with 10% fetal bovine serum (FBS) (Gibco), 0.005 mg/mL insulin (Sigma‐Aldrich, USA), 0.005 mg/mL transferrin, 100 ng/mL hydrocortisone (Sigma‐Aldrich, USA), and 10 ng/mL epidermal growth factor (Sigma‐Aldrich). HT29, RKO, DLD‐1, and HCT 116 cells were maintained in RPMI‐1640 medium containing 10% FBS and 1% penicillin/streptomycin. CACO2 cells were cultured in DMEM plus 10% FBS and 1% penicillin/streptomycin. All cells were kept at 5% CO_2_ and 37°C with regular testing for mycoplasma detection. Subculturing was conducted at 80%–90% confluence, and only low‐passage cells were utilized to maintain experimental reliability.

To pharmacologically inhibit non‐canonical Wnt signalling, cells were treated with the Wnt5a antagonist Box5 (MCE, HY‐123071, USA). Based on previously established protocols [19], cells were incubated with 1 μM Box5 for 1 h prior to subsequent functional assays. This specific concentration and treatment duration were selected according to the literature to ensure effective and specific target inhibition while minimizing potential off‐target cytotoxicity.

### 2.4 Lentivirus Construction and Cell Transfection

Three short hairpin RNAs (shRNAs) targeting GNG10 (5′‐GTGGAGAGGATCAAGGTCTCT‐3′, 5′‐AGCCTAGATCCTGTGCTTTAC‐3′, and 5′‐CAGAGCTTCAACAGTACTGTA‐3′) were synthesized and integrated into a lentiviral backbone. Lentiviral assembly was carried out in HEK293T cells through the delivery of shRNA vectors alongside helper plasmids (psPAX2 and pMD2.G) employing Lipofectamine 3000 (Invitrogen, USA), per the supplier's manual. After 48 h, the virus‐containing broth was harvested, passed through a 0.45 μm filter, and preserved at −80°C until needed. To achieve lentiviral transduction, HCT116 and RKO cells were plated in 6‐well plates at 3 × 10^5^ cells per well and exposed to lentivirus (MOI = 10) supplemented with 8 μg/mL polybrene. After 24 h, the culture fluid was exchanged for new complete growth medium. Infected cells were isolated using 2 μg/mL puromycin for 72 h to produce GNG10‐silenced stable lines. The success of depletion was validated through quantitative real‐time PCR (qRT‐PCR) and western blotting.

### 2.5 Quantitative Real‐Time PCR (qPCR)

Isolation of total RNA from cells used TRIzol reagent (Sigma, USA) per the manufacturer's instructions. A NanoDrop spectrophotometer (Thermo Fisher Scientific) was used to determine RNA concentration and purity. Synthesis of cDNA utilized Hiscript QRT Supermix for qPCR (+gDNA Wiper) via reverse transcription. The AceQ qPCR SYBR Green Master Mix was used for qPCR on an ABI VII7 Real‐Time PCR system (ABI, USA). Reaction mixtures (10 μL total volume) contained 5.0 μL SYBR Green Master Mix, 0.25 μL of each primer (10 μM), 0.2 μL Dye2, 2.0 μL cDNA, and 2.3 μL RNase‐Free H_2_O. Thermal cycling included initial denaturation at 95°C for 30 s, followed by 40 cycles of 95°C for 5 s and 60°C for 30 s. To ensure reaction specificity, melt curve analysis followed the amplification process. The 2^−ΔΔCt^ method was used to determine relative gene expression levels, normalized to the GAPDH internal control. The following primer sequences were used for qPCR analysis:

GAPDH: Forward, 5′‐TGACTTCAACAGCGACACCCA‐3′; Reverse, 5′‐CACCCTGTTGCTGTAGCCAAA‐3′.

GNG10: Forward, 5′‐GCTGGCGTGGAGAGGATCAA‐3′; Reverse, 5′‐CAGAGTAAAGCACAGGATCTAGGC‐3′.

RHOA: Forward, 5′‐TGGAAAGCAGGTAGAGTTGGC‐3′; Reverse, 5′‐ACATCGGTATCTGGGTAGGAGA‐3′.

JNK: Forward, 5′‐TGGTACACGATGCTTTACAACC‐3′; Reverse, 5′‐CACATGCAATCTTCTTCACCAGA‐3′.

PLCβ3: Forward, 5′‐CGCTGGCTTCACTTCGCATT‐3′; Reverse, 5′‐GATGGCAGAGACAGGCAGGAT‐3′.

NFATc1: Forward, 5′‐TGTGCCGGAATCCTGAAACTC‐3′; Reverse, 5′‐GAGCATTCGATGGGGTTGGAG‐3′.

### 2.6 Western Blot Analysis

Western blot analysis was performed to evaluate protein expression levels. For total protein extraction from cells, RIPA lysis buffer (Beyotime, China) containing protease and phosphatase inhibitors was used. A BCA Protein Assay Kit (Beyotime, China) was used to measure protein concentration. Protein (20 μg) was separated using 10% SDS‐PAGE and then transferred onto PVDF membranes (Millipore, USA). After blocking with 5% non‐fat milk in TBST for 1 h at room temperature, membranes were incubated at 4°C overnight with primary antibodies. The primary antibodies included: anti‐GNG10 (1:1000, Affinity Biosciences, DF2205), anti‐RHOA (1:1000, Santa Cruz, sc‐16639), anti‐JNK (1:3000, Abcam, ab179461), anti‐NFATC1 (1:1000, Proteintech, 66963‐1‐Ig), anti‐SOX2 (1:500, Santa Cruz, sc‐17320), anti‐Nanog (1:1000, Abcam, 3369‐1), anti‐OCT4 (1:3000, Proteintech, CL488‐11263), anti‐CD44 (1:2000, Proteintech, CL405‐65608), anti‐CD133 (1:1000, Abcam, ab66141), and anti‐GAPDH (1:30,000, Proteintech, 13937‐1‐AP). Following three washes with TBST, membranes were incubated for 1 h at room temperature with horseradish peroxidase (HRP)‐conjugated secondary antibodies, including goat anti‐rabbit IgG (1:3000, Molecular Probes, A‐11008) or goat anti‐mouse IgG (1:3000, Beyotime, P0946). An enhanced chemiluminescence (ECL) detection system (GE AI600, USA) was used to detect protein bands and capture images. ImageJ software was used for densitometric analysis. To normalize protein expression levels, GAPDH served as an internal control.

### 2.7 Cell Counting Kit‐8 (CCK‐8) Assay

A Cell Counting Kit‐8 (CCK‐8) assay (Sigma, USA) was used to assess cell viability. In 96‐well plates, HCT116 and RKO cells were seeded at a density of 2000 cells per well in 100 μL of complete culture medium. For each experimental group, triplicate wells were included. Following incubation for 24 h, 10 μL of the CCK‐8 solution was added to each well followed by 2–4 h of incubation at 37°C. A microplate reader (Tecan Infinite M2009PR, Switzerland) was used to measure optical density (OD) at 450 nm. The OD values were used to calculate cell viability. Three independent repetitions of the experiment were performed.

### 2.8 Flow Cytometry Analysis

Annexin V‐Propidium Iodide (PI) dual staining was used to assess apoptosis. Following collection and two washes with phosphate‐buffered saline (PBS), the cells were resuspended in 1× binding buffer. Each sample was incubated in the dark at room temperature for 15 min after the addition of 5 μL of Annexin V‐APC and 5 μL of PI (eBioscience, USA). Apoptosis analysis was performed using a flow cytometer after adding 1× binding buffer, with at least 10,000 events recorded per sample. FlowJo software was used to process the data.

Cells were harvested and washed twice with cold PBS, then resuspended in 100 μL PBS containing 2% FBS for CD44 + CD133+ cell population analysis. In the dark at 4°C, the cells were incubated for 30 min with APC‐conjugated anti‐CD44 antibody (BD Biosciences, USA) and PE‐conjugated anti‐CD133 antibody (Miltenyi Biotec, Germany). Following staining, 500 μL PBS was used to resuspend the washed cells for flow cytometry analysis. FlowJo software was used for data analysis.

### 2.9 Colony Formation Assay

Cells were seeded into 6‐well plates (400–1000 cells/well) and cultured at 37°C with 5% CO_2_ for 14 days. The medium was changed every 3 days. Following incubation, cells were subjected to fixation with 4% paraformaldehyde (Guoyao Chemical Reagent Co., China) for 30 min followed by staining with Giemsa solution (Shanghai Dingguo Biotech, China) for 15 min. Washing with ddH2O and air‐drying the colonies was followed by counting under a microscope.

### 2.10 Wound Healing Assay

Cell migration was assessed using a wound healing assay. Cells were seeded into six‐well plates and cultured in complete medium until approximately 90% confluence was reached. A scratch in the cell monolayer was created using a sterile 200‐μL pipette tip. Washing twice with PBS removed the detached cells, followed by incubation in serum‐free medium. Using an inverted microscope, images of the scratched area were captured at 0 h and selected time points. The closure of the scratched area over time was measured to quantify the migration rate.

### 2.11 Transwell Migration Assay

To conduct migration assays, Transwell chambers (Corning, USA) were utilized. Briefly, 1 × 10^5^–2 × 10^5^ cells suspended in 100 μL of serum‐free medium were seeded into the upper chamber, while the lower chamber was filled with 600 μL of medium containing 30% FBS. After incubation at 37°C for 4–24 h, migrated cells were fixed, stained, and imaged using a microscope (IX73, Olympus, Japan) following the removal of non‐migrated cells. ImageJ software was employed to quantify the number of migrated cells.

### 2.12 3D Tumoursphere Formation Assay

Ultra‐low attachment 6‐well plates (Corning, USA) were used to seed cells at a density of 1 × 10^3^ cells/well for cultivation in serum‐free DMEM/F12 medium supplemented with 20 ng/mL EGF, 20 ng/mL bFGF, and B27 (Gibco, USA). Every 3 days, the culture medium was refreshed. After incubating for 7–14 days at 37°C and 5% CO_2_, a microscope was utilized to image and count the tumourspheres.

### 2.13 Subcutaneous Xenograft Tumour Model

For the establishment of a subcutaneous xenograft tumour model, 4‐week‐old female NCG mice (GemPharmatech) were maintained under pathogen‐free conditions with controlled temperature and humidity. A 200 μL mixture of PBS and Matrigel (Corning, USA) containing RKO cells (1 × 10^7^) was injected subcutaneously into the right flank of each mouse. The volume of tumours was determined every 3 days via the formula: volume = (length × width^2^)/2, to monitor growth. Following 18 days, mice were euthanized via CO_2_ asphyxiation; subsequently, tumours were excised, weighed, and photographed. For further histological analysis, 4% paraformaldehyde (Sigma) was used to fix the tumour tissues. The Medical Ethics Committee of Zhujiang Hospital, Southern Medical University approved all animal experiments, which were carried out in compliance with ethical guidelines.

### 2.14 Statistical Analysis

GraphPad Prism 8.0 (GraphPad Software, USA) and SPSS 26.0 (IBM, USA) were used to perform all statistical analyses. Data obtained from at least three independent experiments are presented as mean ± standard deviation (SD). To compare two groups, an unpaired two‐tailed Student's *t*‐test was employed. One‐way analysis of variance (ANOVA) followed by Tukey's post hoc test was used for multiple group comparisons. The Kaplan–Meier method was utilized for survival analysis, with differences evaluated via the log‐rank test. The chi‐square test (*χ* ^2^ test) was performed for correlation analyses between categorical variables, while continuous variables were analyzed using Spearman's correlation analysis. A value of *p* < 0.05 was considered to indicate statistical significance.

## 3 Results

### 3.1 GNG10 Is Upregulated in CRC and Correlates With Poor Prognosis

**FIGURE 1:**
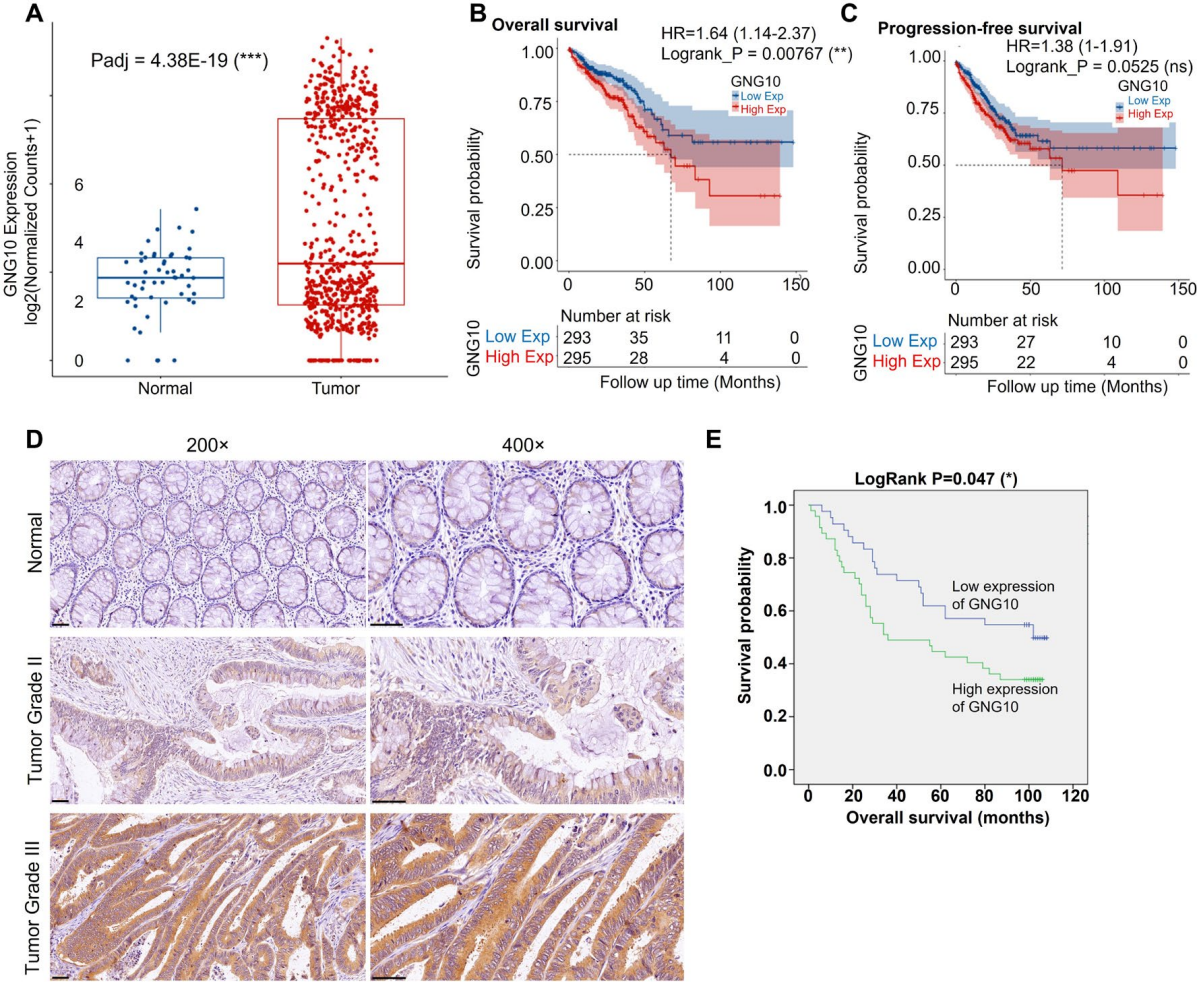
Expression pattern and prognostic implication of GNG10 in colorectal cancer. (A) Transcriptomic analysis from the TCGA dataset reveals the expression pattern of GNG10 in colorectal cancer tissues compared to normal tissues. (B) Kaplan–Meier survival analysis evaluates the relationship between GNG10 expression and overall survival in colorectal cancer patients. (C) The association between GNG10 expression and progression‐free survival in colorectal cancer is further assessed using Kaplan–Meier analysis. (D) Representative immunohistochemical images illustrate GNG10 expression in normal colorectal tissues and in tumour samples of varying histological grades. (E) Prognostic value of GNG10 expression is analysed based on tissue microarray data, using overall survival as the clinical endpoint. Data are presented as mean ± standard deviation (SD). Statistical significance is indicated as follows: *p < 0.05, **p < 0.01, ***p < 0.001, ns, no significance.

For identifying potential regulatory molecules in CRC, bioinformatics analysis was performed using TCGA gene expression data. Of the differentially expressed genes, GNG10 showed significantly higher expression in tumour tissues relative to normal tissues (Figure 1A). Further survival analysis indicated that high GNG10 expression was strongly associated with reduced overall survival (OS) (Figure 1B) and shorter progression‐free survival (PFS) (Figure 1C), implying its potential involvement in CRC progression. To validate these findings at the protein level, we performed immunohistochemical (IHC) staining on a tissue microarray consisting of 89 tumour tissues and 71 normal tissues. Representative images revealed that GNG10 levels were notably elevated in tumour tissues compared to normal tissues (Figure 1D). Quantification of IHC staining scores verified that GNG10 was significantly overexpressed across the CRC samples (Table 1). Furthermore, correlation analysis between GNG10 expression and clinicopathological features indicated a significant association between high GNG10 expression and advanced tumour grade, with higher grade tumours exhibiting stronger GNG10 staining (Table 2 and Figure 1D). Given the clinical heterogeneity of CRC, we further performed multivariate Cox proportional hazards regression analyses to determine whether GNG10 could serve as an independent prognostic factor. We constructed two models to adjust for established clinicopathological features: Model A (adjusting for GNG10, Age, Sex, and overall AJCC Stage) and Model B (adjusting for GNG10, Age, Sex, T Stage, N Stage, and M Stage). As shown in Figure S1, advanced age and advanced tumour stages (e.g., AJCC Stage III/IV, N2, M1) emerged as significant independent risk factors. However, GNG10 expression did not retain statistical significance as an independent prognostic factor in either Model A (*p* = 0.644) or Model B (*p* = 0.400). These data suggest that the prognostic value of GNG10 observed in univariate analysis is closely associated with tumour stage progression. Additionally, survival analysis using follow‐up data from the tissue microarray cohort demonstrated that patients with higher GNG10 expression had significantly worse survival outcomes (Figure 1E), consistent with TCGA‐based bioinformatics analysis. Collectively, these findings suggest that GNG10 may play a crucial role in CRC progression and may serve as a potential prognostic biomarker.

**TABLE 1:** Expression patterns of GNG10 in colorectal cancer tissues and normal tissues revealed in immunohistochemistry analysis.

GNG10 expression	Tumour tissue		Normal tissue	
	Cases	Percentage	Cases	Percentage
Low	42	47.2	68	95.8
High	47	52.8	3	4.2

*Note:* *p* < 0.001.

**TABLE 2:** Relationship between GNG10 expression and tumour characteristics in patients with colorectal cancer.

Features	No. of patients	GNG10 expression		*p*
		Low	High	
All patients	89	42	47	
Gender				0.618
Male	47	21	26	
Female	42	21	21	
Tumour size				0.602
< 5.5 cm	44	22	22	
≥ 5.5 cm	45	20	25	
Grade				< 0.001
II	49	33	16	
III	40	9	31	
Stage				0.609
I	5	3	2	
II	49	23	26	
III	33	16	17	
IV	2	0	2	
T infiltrate				0.132
T1	1	0	1	
T2	5	4	1	
T3	70	34	36	
T4	13	4	9	
Lymphatic metastasis (N)				0.823
N0	54	26	28	
N1	35	16	19	
Metastasis				0.179
M0	87	42	45	
M1	2	0	2	
Serous interstitial expression of PDL1				0.243
> 10%	45	24	21	
≤ 10%	44	18	26	

### 3.2 Knockdown of GNG10 Suppresses CRC Cell Proliferation, Migration, and Promotes Apoptosis

**FIGURE 2:**
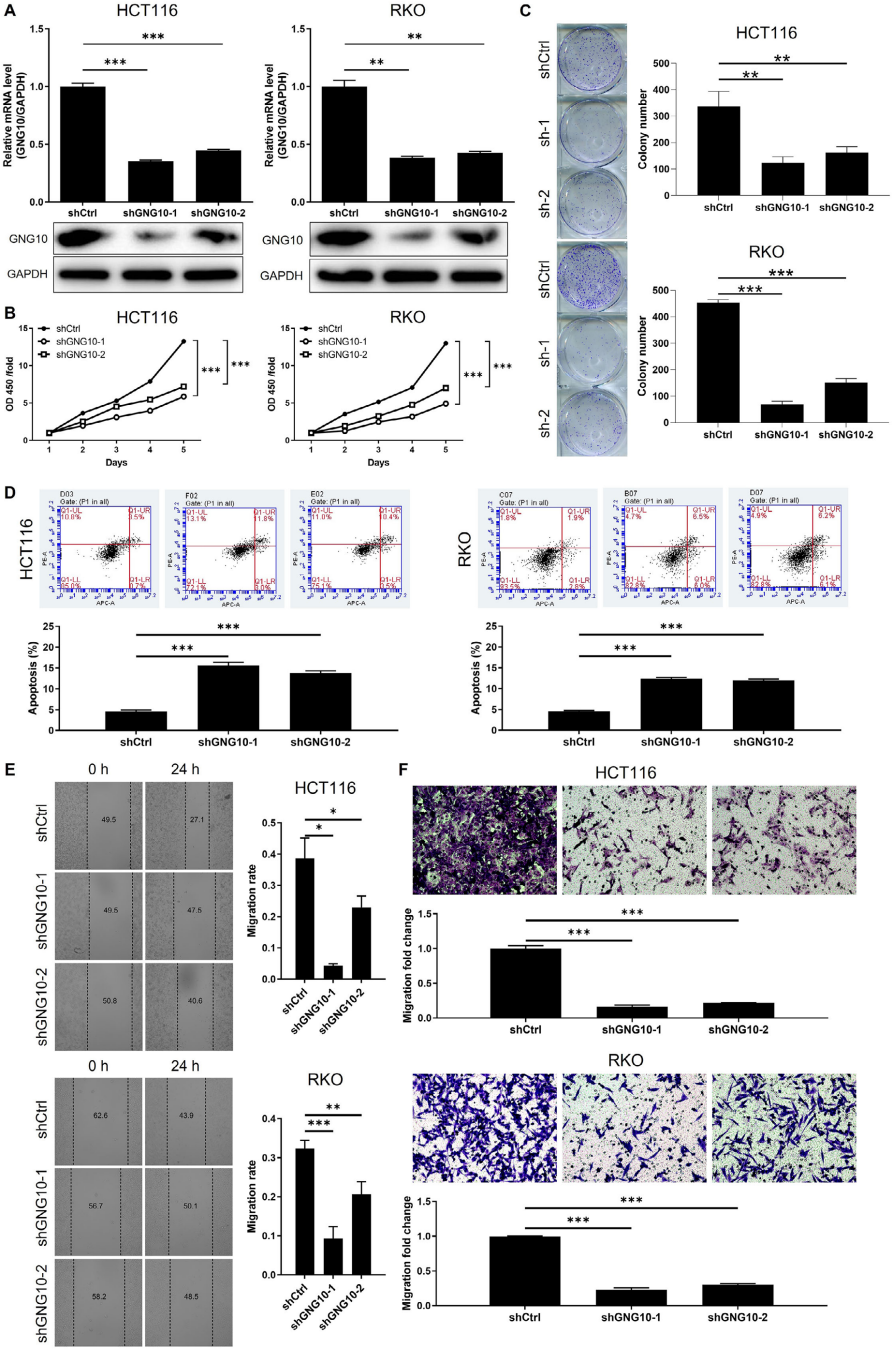
Functional characterization of GNG10 knockdown in colorectal cancer cells. (A) Quantitative PCR and Western blotting are used to confirm the knockdown efficiency of GNG10 in HCT116 and RKO colorectal cancer cell lines. (B) The CCK8 assay is performed to assess the proliferation ability of GNG10‐knockdown cells compared to control cells. (C) A colony formation assay is conducted to evaluate the long‐term proliferative capacity of colorectal cancer cells after GNG10 knockdown. (D) Flow cytometry is employed to quantify apoptotic cell populations in GNG10‐knockdown and control groups. (E) A wound healing assay is used to measure the migration ability of colorectal cancer cells following GNG10 knockdown. (F) Transwell migration assay is performed to further assess the effect of GNG10 knockdown on colorectal cancer cell motility. Data are presented as mean ± standard deviation (SD). Statistical significance is indicated as follows: *p < 0.05, **p < 0.01, ***p < 0.001.

In vitro loss‐of‐function experiments were performed to further elucidate the functional role of GNG10 in CRC progression. Its basal expression was first assessed across a panel of human CRC cell lines. Among the tested lines, HCT116 and RKO cells were found to exhibit the highest endogenous expression levels (Figure S2). Two independent shRNAs were designed to knock down GNG10 expression in CRC cell lines (HCT116 and RKO) to minimize off‐target effects. The qPCR and WB were utilized to validate knockdown efficiency, which confirmed a significant reduction in the shGNG10 groups (Figure 2A). Subsequently, functional assays were performed to assess how GNG10 knockdown affects CRC cell phenotypes. GNG10 knockdown significantly inhibited CRC cell growth, as consistently shown by CCK‐8 proliferation (Figure 2B) and colony formation assay (Figure 2C). Further, flow cytometry analysis revealed that the proportion of apoptotic cells was markedly increased by GNG10 knockdown (Figure 2D), suggesting its role in apoptosis regulation. Additionally, the effects of GNG10 on CRC cell migration were examined. As shown in the wound healing assay (Figure 2E), the closure of the scratch area was significantly delayed in GNG10‐silenced HCT116 and RKO cells compared to the control group (shCtrl) after 24 h of incubation. Consistently, the transwell migration assay (Figure 2F) revealed that GNG10 knockdown led to a dramatic reduction in the number of cells migrating to the lower chamber. Quantitative analysis confirmed that the migration rate and the fold change in migration were significantly lower in the shGNG10‐1 and shGNG10‐2 groups than in the shCtrl group across both cell lines. These results collectively indicate that GNG10 plays a critical role in promoting the malignant phenotypes of CRC cells in vitro.

### 3.3 GNG10 Regulates CRC Progression via the Non‐Canonical Wnt Pathway

**FIGURE 3:**
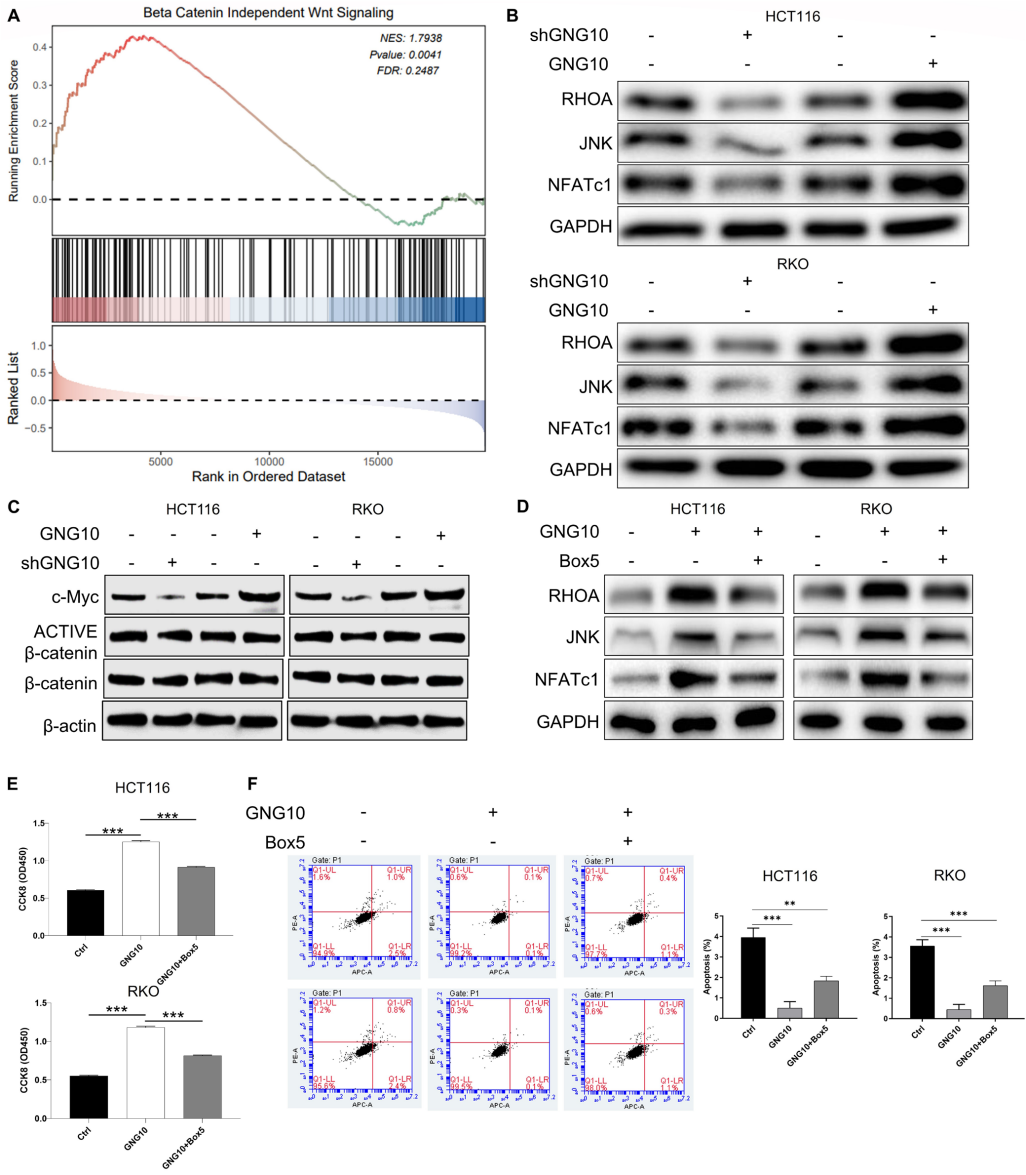
Investigation of the non‐canonical wnt pathway as a downstream effector of GNG10 in colorectal cancer cells. (A) Gene Set Enrichment Analysis (GSEA) is conducted based on TCGA data to identify signalling pathways associated with GNG10 expression, with a focus on the non‐canonical Wnt pathway. (B) Western blotting is used to detect the expression of RHOA, JNK, and NFATc1 in colorectal cancer cells after GNG10 knockdown or overexpression. (C) Western blot analysis of canonical Wnt signalling markers (Active β‐catenin and Total β‐catenin) and downstream target c‐Myc in HCT116 and RKO cells following GNG10 knockdown (shGNG10) or overexpression (GNG10‐OE). β‐Actin was used as a loading control. (D) Western blot analysis is performed to evaluate the effect of Box5, a non‐canonical Wnt pathway inhibitor, on the expression of RHOA, JNK, and NFATc1 in GNG10‐overexpressing cells. (E) The CCK‐8 assay is used to assess colorectal cancer cell proliferation following Box5 treatment in GNG10‐overexpressing cells. (F) Flow cytometry is employed to evaluate apoptosis in GNG10‐overexpressing colorectal cancer cells with or without Box5 treatment. Data are presented as mean ± standard deviation (SD). Statistical significance is indicated as follows: **P < 0.01, ***p < 0.001.

To investigate the downstream molecular mechanisms of GNG10 in CRC, Gene Set Enrichment Analysis (GSEA) was performed based on TCGA data. The GSEA results (Figure 3A) revealed that GNG10 expression was positively enriched in the ‘β‐catenin Independent Wnt Signaling’ pathway, suggesting GNG10 may mediate non‐canonical Wnt signalling. To validate this hypothesis, the expression levels of key components in this pathway were examined by WB after modulating GNG10 expression. In both HCT116 and RKO cells, GNG10 knockdown led to a significant decrease in the protein levels of RHOA, JNK, and NFATc1 (Figure 3B). Conversely, GNG10 overexpression markedly upregulated the expression of these proteins. To rigorously exclude the involvement of the canonical Wnt cascade and confirm pathway specificity, we further evaluated β‐catenin expression. As shown in Figure 3C, neither GNG10 knockdown nor overexpression altered the levels of Active (non‐phosphorylated) or Total β‐catenin. Furthermore, the downstream target c‐Myc was upregulated by GNG10 in a strictly β‐catenin‐independent manner, likely driven by the aforementioned non‐canonical JNK cascade. These data conclusively demonstrate that GNG10 specifically activates the non‐canonical Wnt signalling axis in CRC. To further assess the functional relevance of the non‐canonical Wnt pathway in GNG10‐mediated regulation of CRC cells, we treated GNG10‐overexpressing cells with Box5, a specific inhibitor of this pathway. Box5 treatment reversed the upregulation of RHOA, JNK, and NFATc1 induced by GNG10 overexpression (Figure 3D), confirming the dependency of these molecular changes on non‐canonical Wnt signalling. More importantly, Box5 treatment not only suppressed GNG10‐induced activation of the non‐canonical Wnt pathway but also significantly attenuated GNG10 overexpression‐mediated enhancement of cell proliferation (Figure 3E) and suppression of apoptosis (Figure 3F). These findings indicated that the non‐canonical Wnt pathway is a key downstream mediator of GNG10 in CRC progression, highlighting its potential as a therapeutic target for GNG10‐driven tumourigenesis.

### 3.4 GNG10 Regulates CRC Stemness via the Wnt Pathway

**FIGURE 4:**
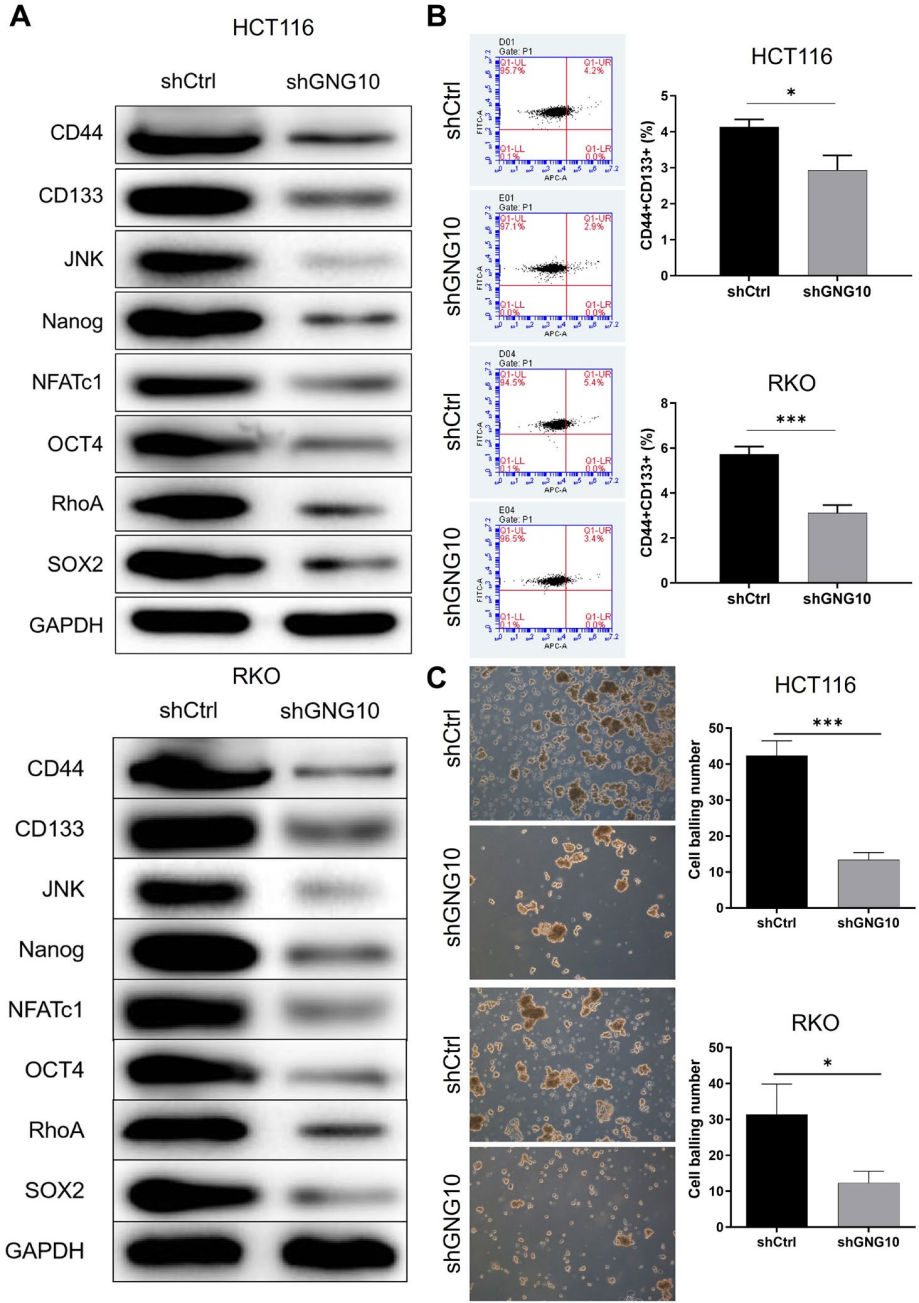
Evaluation of colorectal cancer stemness following GNG10 knockdown. (A) Western blotting is performed to examine the expression of stemness‐associated markers CD44, CD133, OCT4, Nanog, and SOX2 in colorectal cancer cells after GNG10 knockdown. (B) Flow cytometry is used to analyse the proportion of CD44^+^CD133^+^ cell populations in colorectal cancer cells with or without GNG10 knockdown. (C) A 3D tumour sphere formation assay is conducted to assess the self‐renewal capacity of colorectal cancer cells following GNG10 knockdown. Data are presented as mean ± standard deviation (SD). Statistical significance is indicated as follows: *p < 0.05, ***p < 0.001.

Given the association between non‐canonical Wnt signalling and cancer stemness, we further investigated whether GNG10 modulates the cancer stem cell (CSC)‐like properties of CRC cells. Western blot analysis (Figure 4A) demonstrated that the protein levels of key stemness markers, including CD44, CD133, OCT4, NANOG, and SOX2, were significantly downregulated in CRC cells following GNG10 knockdown. Consistently, the protein levels of non‐canonical Wnt pathway components (JNK, NFATc1, and RhoA) were also reduced, further supporting the link between GNG10‐mediated signalling and stemness maintenance. To further validate this finding, we performed flow cytometry to examine the proportion of CD44^+^CD133^+^ cells, a well‐established marker combination for cancer stem‐like cells. As shown in Figure 4B, GNG10 silencing led to a significant decrease in the CD44^+^CD133^+^ cell proportion in both HCT116 and RKO cell lines. Furthermore, tumoursphere formation assays were conducted to evaluate the self‐renewal capacity of these cells. We observed that GNG10 knockdown markedly inhibited the number and size of tumourspheres formed by CRC cells compared to the control group (Figure 4C). Collectively, these data indicate that GNG10 is involved in maintaining CRC cell stemness, possibly through the activation of the non‐canonical Wnt signalling pathway.

### 3.5 Non‐Canonical Wnt Pathway Mediates GNG10‐Induced Cancer Stemness in CRC

**FIGURE 5:**
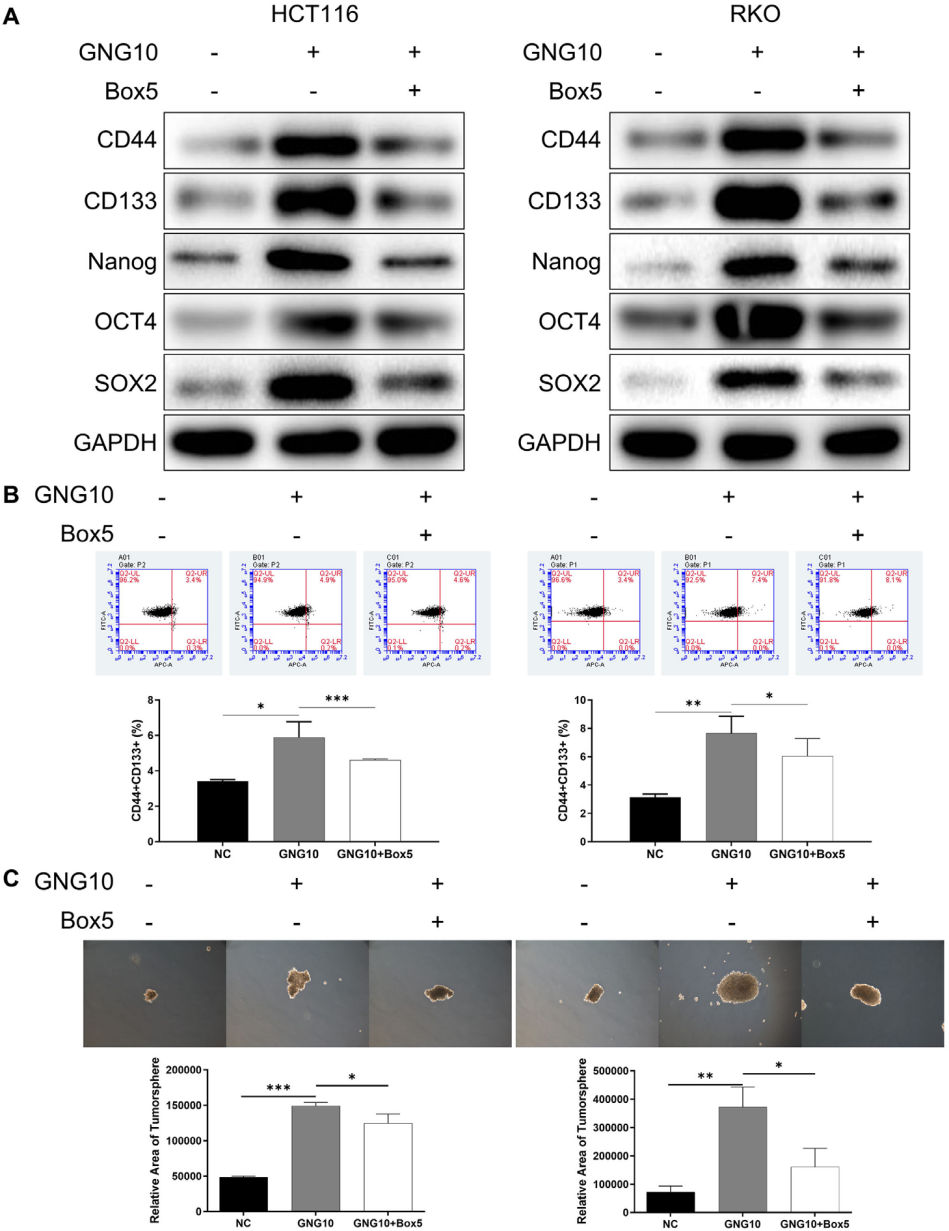
Assessment of the role of the non‐canonical Wnt pathway in GNG10‐induced cancer stemness in colorectal cancer cells. (A) Western blotting is performed to evaluate the expression of stemness‐associated markers CD44, CD133, Nanog, OCT4, and SOX2 in colorectal cancer cells following GNG10 overexpression and treatment with the Wnt pathway inhibitor Box5. (B) Flow cytometry is used to analyse the proportion of CD44^+^CD133^+^ cell populations under GNG10 overexpression and Box5 treatment conditions. (C) A 3D tumour sphere formation assay is conducted to assess the self‐renewal ability of colorectal cancer cells after GNG10 overexpression and subsequent Box5 treatment. Data are presented as mean ± standard deviation (SD). Statistical significance is indicated as follows: *p < 0.05, **p < 0.01, ***p < 0.001.

To determine whether the non‐canonical Wnt pathway mediates the regulatory role of GNG10 in CRC stemness, we initially performed a pharmacological rescue experiment by overexpressing GNG10 and subsequently treating the cells with Box5, a specific inhibitor of the non‐canonical Wnt pathway. As shown by western blot analysis (Figure 5A), GNG10 overexpression‐induced upregulation of stemness‐related markers, including CD44, CD133, Nanog, OCT4, and SOX2, was significantly attenuated by the addition of Box5. Consistently, flow cytometry analysis (Figure 5B) revealed that the enrichment of the CD44^+^CD133^+^ cell population caused by GNG10 overexpression was markedly reversed following Box5 treatment. Furthermore, tumoursphere formation assays (Figure 5C) were conducted to assess the functional impact on self‐renewal. GNG10 overexpression significantly increased the size and relative area of tumourspheres, but this effect was effectively counteracted by Box5‐mediated pathway inhibition.

**FIGURE 6:**
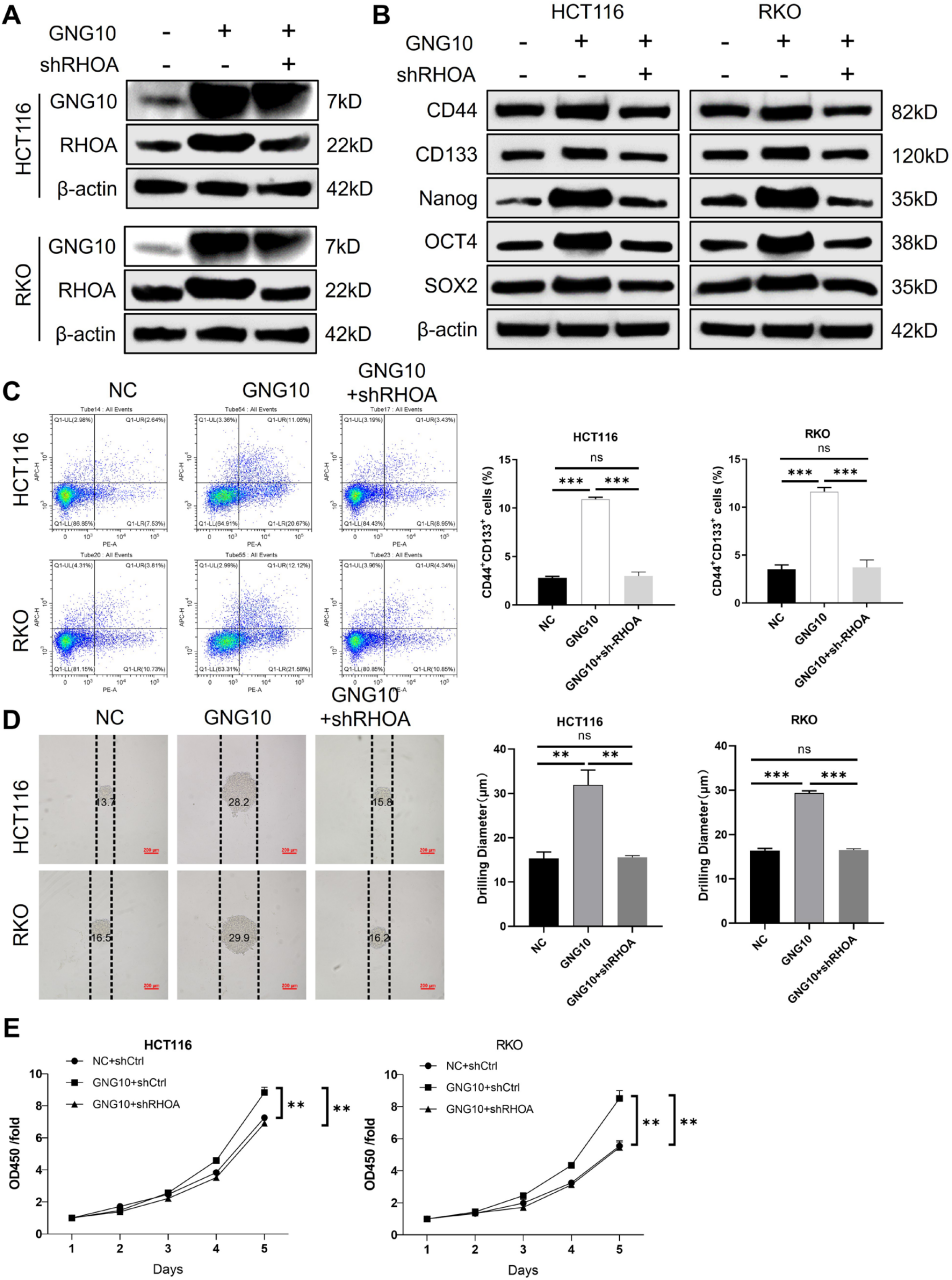
Genetic ablation of RHOA rescues GNG10‐induced cancer stemness and malignant phenotypes in CRC cells. (A) Western blot validation of GNG10 overexpression and concurrent RHOA knockdown (shRHOA) in HCT116 and RKO cells. β‐actin served as the loading control. (B) Western blot analysis of key cancer stem cell markers (CD44, CD133, Nanog, OCT4, and SOX2) in the indicated groups. (C) Flow cytometry evaluation of the CD44+/CD133+ CSC subpopulation. (D) Tumoursphere formation assays evaluating self‐renewal capacity. Representative images (left) and quantification of tumoursphere drilling diameters (right) show that RHOA depletion attenuated GNG10‐enhanced sphere formation. Scale bars: 200 μm. (E) CCK‐8 assays demonstrating that RHOA knockdown significantly reversed the proliferative advantage conferred by GNG10 overexpression. Data are presented as mean ± SD. Statistical significance is indicated as follows: **P < 0.01, ***p < 0.001.

While pharmacological inhibition strongly suggested the involvement of the non‐canonical Wnt pathway, reliance on a single small‐molecule inhibitor risks potential off‐target effects. To definitively establish causality, we performed a concurrent genetic rescue experiment by targeting RHOA, a critical downstream effector of this cascade. Western blot confirmed the successful overexpression of GNG10 alongside efficient RHOA depletion (Figure 6A). Remarkably, genetic ablation of RHOA (shRHOA) completely phenocopied the effects of Box5 treatment. Specifically, RHOA knockdown robustly abrogated the GNG10‐induced upregulation of all evaluated CSC markers (CD44, CD133, Nanog, OCT4, and SOX2) (Figure 6B) and entirely reversed the expansion of the CD44+/CD133+ subpopulation (Figure 6C). Furthermore, the enhanced tumoursphere formation (Figure 6D) and accelerated proliferation (Figure 6E) conferred by GNG10 were significantly attenuated upon RHOA depletion. Collectively, the concordant results from both pharmacological inhibition and genetic modulation unequivocally demonstrate that the non‐canonical Wnt pathway, specifically the RHOA‐mediated axis, plays a crucial role in mediating GNG10‐induced CRC stemness, further supporting its involvement in tumour progression.

### 3.6 GNG10 Knockdown Suppresses Tumour Growth and Cancer Stemness In Vivo

**FIGURE 7:**
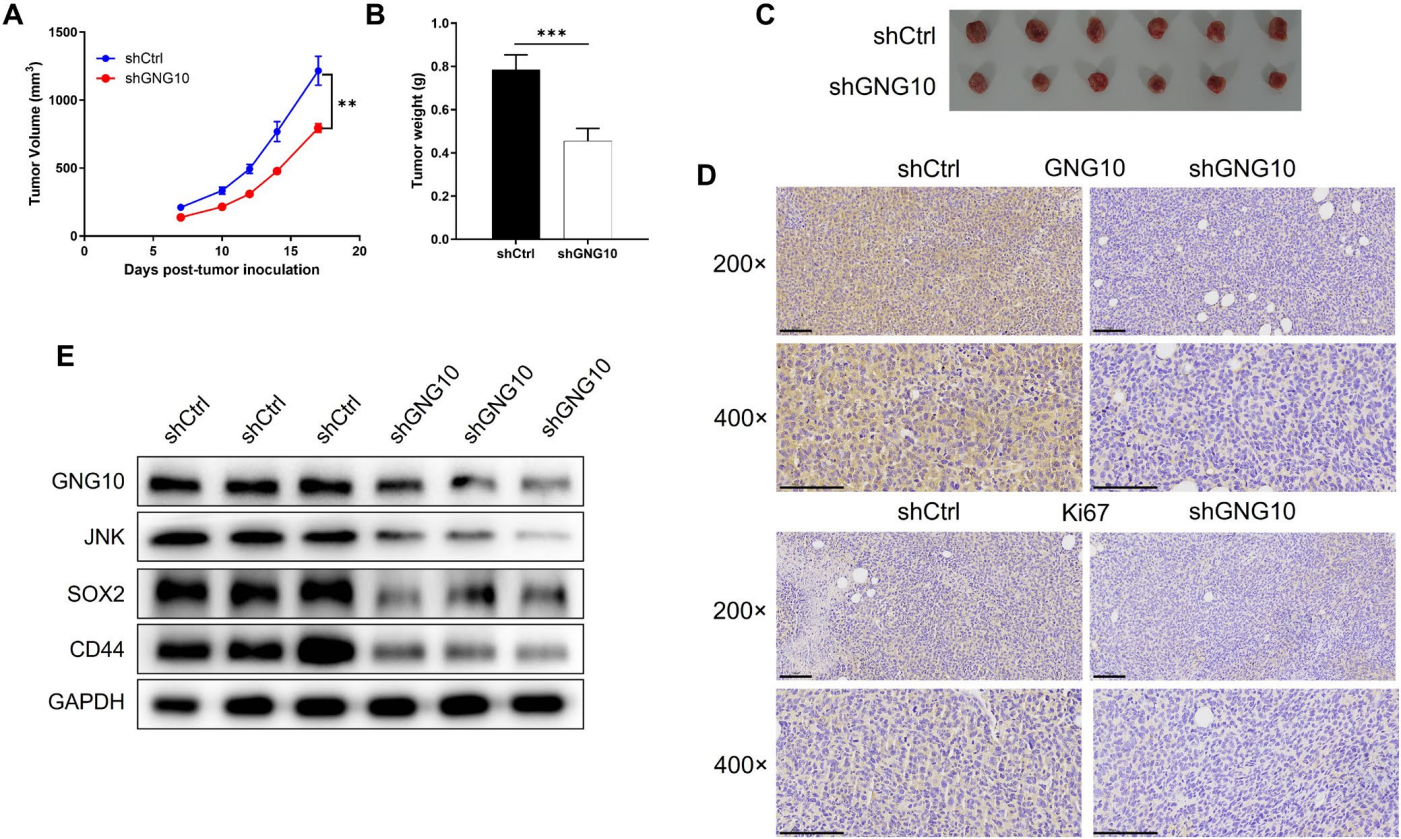
In vivo evaluation of tumour growth and stemness following GNG10 knockdown in a colorectal cancer xenograft model. (A) Tumour volumes are measured at multiple time points (days 7, 10, 12, 14, and 17) in mice injected subcutaneously with control or GNG10‐knockdown (shGNG10) colorectal cancer cells. (B) Final tumour weights are recorded at the experimental endpoint to compare tumour burden between the control and shGNG10 groups. (C) Representative photographs of tumours excised from mice injected with control or shGNG10 cells are shown to illustrate differences in tumour size. (D) Immunohistochemical staining is performed to assess the expression levels of GNG10 and the proliferation marker Ki67 in xenograft tumour tissues. (E) Western blotting is used to examine the expression of JNK, SOX2, and CD44 in tumour tissues derived from control and GNG10‐knockdown groups. Data are presented as mean ± standard deviation (SD). Statistical significance is indicated as follows: **p < 0.01, ***p < 0.001.

To further validate the role of GNG10 in CRC progression, we established a subcutaneous xenograft model in NCG mice. The tumour volume was measured at multiple time points (days 7, 10, 12, 14, and 17) following tumour cell implantation. The results demonstrated that tumours derived from GNG10‐knockdown (shGNG10) cells exhibited significantly reduced growth compared to the control group (Figure 7A). Consistently, tumour weight measurement at the experimental endpoint confirmed that GNG10 knockdown significantly suppressed the tumour burden (Figure 7B). Additionally, images of excised tumours provided a visual representation of reduced tumour size in the shGNG10 group (Figure 7C). Immunohistochemical (IHC) staining further confirmed lower GNG10 expression in the shGNG10 group, validating the efficiency of knockdown in vivo. Notably, Ki67 staining, a marker of cell proliferation, was significantly reduced in GNG10‐knockdown tumours, which is consistent with our in vitro findings that GNG10 promotes CRC cell proliferation (Figure 7D). Furthermore, WB analysis of xenograft tumour tissues revealed that GNG10 knockdown suppressed the expression of key proteins associated with the non‐canonical Wnt pathway (JNK) and cancer stemness markers (SOX2 and CD44) (Figure 7E). These findings indicate strong evidence that GNG10 promotes CRC tumour growth and stemness through the non‐canonical Wnt signalling pathway, reinforcing its potential as a therapeutic target.

## 4 Discussion

GNG10 belongs to the G protein gamma subunit family and has been linked to various biological processes, ranging from haematopoiesis to vascular remodelling and tissue regeneration. Its significant upregulation in bone marrow‐derived erythroid progenitors [20], coupled with its status as a hub gene in IPAH [21] and liver regeneration [13], strongly suggests that GNG10 is a key driver of cellular differentiation and proliferative capacity. In addition to its physiological processes, GNG10 has been linked to multiple cancers. It has been identified as a frequently mutated gene in melanoma [22] and a prognostic factor in gliomas through the PI3K‐Akt pathway [12]. In nasopharyngeal carcinoma, it is associated with radiotherapy response and survival outcomes [14], reinforcing its potential role in tumour progression and therapy resistance. Furthermore, GNG10 was found to distinguish latent from active tuberculosis [23] and was identified as a possible interaction partner of cryptochrome 4 (Cry4) in migratory birds, suggesting additional functions in immune regulation and sensory signalling [24].

Building on this prior knowledge, our study provides direct evidence that GNG10 functions as an oncogene in CRC. We demonstrated that GNG10 was significantly overexpressed in CRC tumour tissues, and that its elevated expression was correlated with poor prognosis in univariate analyses. However, it is important to note a clinical limitation: in our subsequent multivariate Cox regression models, GNG10 did not retain its significance as an independent prognostic factor when adjusted for established clinicopathological parameters such as overall AJCC and TNM stages. We interpret this loss of independent prognostic value as an indication that GNG10 overexpression is deeply intertwined with the malignant progression of the tumour. Consequently, its predictive power is largely subsumed by macroscopic advanced pathological staging. Despite these challenges, recent large‐scale studies have demonstrated that combining transcriptomic signatures with sophisticated machine learning algorithms can yield robust prognostic indices that outperform traditional parameters, providing a more nuanced understanding of cancer survival and therapeutic response [25]. Although this may limit its utility as a standalone clinical prognostic biomarker, its significant upregulation in advanced CRC strongly underscores a vital biological role in driving tumour malignancy. Functional assays further supported this, demonstrating that GNG10 knockdown suppressed CRC cell proliferation and migration, while promoting apoptosis, indicating its indispensable role in tumour progression.

As a fundamental pathway governing development and homeostasis, Wnt signalling is frequently co‐opted in cancer development. In CRC, deregulated Wnt activity contributes to tumour initiation, progression, and therapy resistance [26, 27]. While the canonical Wnt/β‐catenin pathway has a well‐defined oncogenic role, the non‐canonical pathway has emerged as an equally important modulator of tumour behaviour, influencing cell proliferation, migration, invasion, and stemness‐like traits [27]. Rather than stabilizing β‐catenin, non‐canonical Wnt signals through RHOA, JNK, and NFATc1 to drive plasticity and disease advancement [28].

Among the fundamental traits of malignant progression, the presence of cancer stem cells (CSCs) is critical. This specialized cell fraction exhibits robust self‐renewal, resistance to treatment, and enhanced tumorigenic capacity [28, 29]. Wnt signalling, particularly the non‐canonical Wnt pathway, is a critical regulator of CSC maintenance [28, 30, 31]. Research has established that Wnt pathway activation drives stem cell‐like traits in cancer by upregulating core transcription factors, including OCT4, SOX2, and NANOG. Conversely, blocking Wnt signalling restricts tumour expansion by depleting the CSC pool [30, 31]. The interaction between Wnt signalling and metabolic reprogramming further enhances CSC properties, contributing to tumour progression and metastasis [29].

Herein, GNG10 was identified as an oncogenic gene in CRC progression and demonstrated its functional association with the noncanonical Wnt pathway. Bioinformatic analysis and in vitro experiments suggested that GNG10 overexpression activated the RHOA, JNK, and NFATc1 signalling. Crucially, we confirmed pathway specificity by demonstrating that GNG10 manipulation did not alter the protein levels of active or total β‐catenin, thereby strictly excluding the canonical Wnt/β‐catenin cascade in GNG10‐mediated oncogenesis. Further functional assays confirmed that GNG10 overexpression promoted CRC cell proliferation, migration, and stemness, whereas its knockdown suppressed these malignant phenotypes. Importantly, while pharmacological inhibition of the non‐canonical Wnt pathway using Box5 reversed the oncogenic effects of GNG10, we further established a definitive causal relationship through genetic modulation. Targeted genetic ablation of the downstream effector RHOA (shRHOA) completely phenocopied the effects of Box5, robustly abrogating GNG10‐induced cancer stemness and proliferative advantages. Together, this dual validation provides strong evidence that GNG10 regulates CRC progression specifically through the RHOA‐dependent non‐canonical Wnt signalling.

Moreover, our in vivo results validated that GNG10 knockdown significantly suppressed tumour growth, reduced CSC markers (CD44 and CD133), and inhibited the activation of Wnt‐related downstream effectors (JNK and SOX2). These findings are consistent with previous studies demonstrating that Wnt signalling is essential for maintaining CSC populations and promoting CRC progression [31, 32].

Despite the robust evidence presented, we acknowledge certain limitations. First, while strongly associated with poor patient survival in univariate analyses, GNG10 did not retain independent prognostic significance in multivariate Cox regression models when adjusted for macroscopic parameters such as overall AJCC and TNM stages, suggesting its predictive power is closely intertwined with advanced pathological progression. Second, while we firmly established the downstream involvement of the non‐canonical Wnt pathway using both pharmacological (Box5) and genetic (shRHOA) rescue approaches, the direct upstream mechanistic link—specifically, how GNG10 physically interacts with Wnt receptors or upstream mediators to initiate this cascade—remains to be elucidated. Future studies aimed at identifying the direct binding partners of GNG10 will be critical for fully unlocking its therapeutic potential.

Overall, our study provides compelling evidence that GNG10 functions as an oncogene in CRC by activating the noncanonical Wnt pathway and enhancing cancer cell stemness. Given the established role of Wnt signalling in therapy resistance and tumour recurrence, inhibiting GNG10 or its related pathways could provide new therapeutic strategies for CRC. Further investigations into the molecular interactions between GNG10, Wnt signalling, and CSC regulation are critical for understanding its potential as a therapeutic target in CRC and other malignancies.

## Author Contributions

**Ou Li:** data curation. **Yuting Tang:** formal analysis, methodology. **Jianping He:** formal analysis, methodology, writing – review and editing. **Tianlai Liu:** conceptualization, writing – original draft. **Xitao Zhang:** conceptualization, data curation, writing – review and editing. **Xuexiao Li:** data curation. **Yaoqian Liu:** data curation.

## Funding

This study was financially supported by Guangdong Medical Science and Technology Research Foundation (No. B2024106).

## Ethics Statement

The Medical Ethics Committee of Zhujiang Hospital, Southern Medical University approved all animal experiments, which were conducted in accordance with ethical guidelines.

## Consent

The authors have nothing to report.

## Conflicts of Interest

The authors declare no conflicts of interest.

## Supporting information


**Figure S1:** Multivariate Cox proportional hazards regression analysis of GNG10 and clinicopathological characteristics in colorectal cancer. Forest plots displaying the hazard ratios (HRs) and 95% confidence intervals (CIs) for overall survival based on two multivariate models. (A) Model A assesses the prognostic value of GNG10 after adjusting for age, sex, and overall AJCC stage. (B) Model B assesses the prognostic value of GNG10 after adjusting for age, sex, and individual tumour‐node‐metastasis (TNM) stages. Black squares represent the point estimate of the HR, and horizontal lines denote the 95% CIs. The vertical dashed line corresponds to an HR of 1.0 (no effect). Variables with *p* < 0.05 were considered statistically significant. The results indicate that while advanced age and late tumour stages (AJCC Stage III/IV, N2, and M1) serve as significant independent risk factors, GNG10 expression does not retain independent prognostic significance when adjusted for these established macroscopic pathological parameters.
**Figure S2:** Basal endogenous expression of GNG10 across normal and colorectal cancer cell lines. Quantitative real‐time PCR (qRT‐PCR) analysis of baseline GNG10 mRNA expression levels in a normal human colon epithelial cell line (FHC) and five human colorectal cancer (CRC) cell lines (HT29, RKO, DLD‐1, HCT116, and CACO2). GAPDH was used as the internal control for normalization. Data are presented as the mean ± SD from three independent experiments. ***p < 0.001 versus the FHC group.

## Data Availability

Reasonable requests for the data supporting the findings of this study may be directed to the corresponding author.

## References

[1] F. Bray , M. Laversanne , H. Sung , et al., “Global Cancer Statistics 2022: GLOBOCAN Estimates of Incidence and Mortality Worldwide for 36 Cancers in 185 Countries,” CA: A Cancer Journal for Clinicians 74, no. 3 (2024): 229–263. 38572751 10.3322/caac.21834

[2] S. He , C. Xia , H. Li , et al., “Cancer Profiles in China and Comparisons With the USA: A Comprehensive Analysis in the Incidence, Mortality, Survival, Staging, and Attribution to Risk Factors,” Science China. Life Sciences 67, no. 1 (2024): 122–131. 37755589 10.1007/s11427-023-2423-1

[3] R. L. Siegel , T. B. Kratzer , A. N. Giaquinto , H. Sung , and A. Jemal , “Cancer Statistics, 2025,” CA: A Cancer Journal for Clinicians 75, no. 1 (2025): 10–45. 39817679 10.3322/caac.21871 PMC11745215

[4] R. Abedizadeh , F. Majidi , H. R. Khorasani , H. Abedi , and D. Sabour , “Colorectal Cancer: A Comprehensive Review of Carcinogenesis, Diagnosis, and Novel Strategies for Classified Treatments,” Cancer Metastasis Reviews 43, no. 2 (2024): 729–753. 38112903 10.1007/s10555-023-10158-3

[5] V. A. Ionescu , G. Gheorghe , N. Bacalbasa , A. L. Chiotoroiu , and C. Diaconu , “Colorectal Cancer: From Risk Factors to Oncogenesis,” Medicina (Kaunas, Lithuania) 59, no. 9 (2023): 1646. 37763765 10.3390/medicina59091646 PMC10537191

[6] L. Klimeck , T. Heisser , M. Hoffmeister , and H. Brenner , “Colorectal Cancer: A Health and Economic Problem,” Best Practice & Research. Clinical Gastroenterology 66 (2023): 101839. 37852707 10.1016/j.bpg.2023.101839

[7] F. Ciardiello , D. Ciardiello , G. Martini , S. Napolitano , J. Tabernero , and A. Cervantes , “Clinical Management of Metastatic Colorectal Cancer in the Era of Precision Medicine,” CA: A Cancer Journal for Clinicians 72, no. 4 (2022): 372–401. 35472088 10.3322/caac.21728

[8] S. G. Patel , J. J. Karlitz , T. Yen , C. H. Lieu , and C. R. Boland , “The Rising Tide of Early‐Onset Colorectal Cancer: A Comprehensive Review of Epidemiology, Clinical Features, Biology, Risk Factors, Prevention, and Early Detection,” Lancet Gastroenterology & Hepatology 7, no. 3 (2022): 262–274. 35090605 10.1016/S2468-1253(21)00426-X

[9] N. P. Tjader and A. E. Toland , “Immunotherapy for Colorectal Cancer: Insight From Inherited Genetics,” Trends Cancer 10, no. 5 (2024): 444–456. 38360438 10.1016/j.trecan.2024.01.008 PMC11096082

[10] Q. Li , S. Geng , H. Luo , et al., “Signaling Pathways Involved in Colorectal Cancer: Pathogenesis and Targeted Therapy,” Signal Transduction and Targeted Therapy 9, no. 1 (2024): 266. 39370455 10.1038/s41392-024-01953-7 PMC11456611

[11] N. Wang , J. Li , J. He , et al., “Knockdown of lncRNA CCAT1 Inhibits the Progression of Colorectal Cancer via Hsa‐miR‐4679 Mediating the Downregulation of GNG10,” Journal of Immunology Research 2021 (2021): 8930813. 35005034 10.1155/2021/8930813 PMC8739552

[12] B. T. Zhang , P. C. Leung , C. K. Wong , and D. J. Wang , “The Immunomodulatory Effects of Vitamin D on COVID‐19 Induced Glioblastoma Recurrence via the PI3K‐AKT Signaling Pathway,” International Journal of Molecular Sciences 25, no. 23 (2024): 12952. 39684661 10.3390/ijms252312952 PMC11641820

[13] Y. Zhu , Z. Li , J. Zhang , M. Liu , X. Jiang , and B. Li , “Identification of Crucial lncRNAs and mRNAs in Liver Regeneration After Portal Vein Ligation Through Weighted Gene Correlation Network Analysis,” BMC Genomics 23, no. 1 (2022): 665. 36131263 10.1186/s12864-022-08891-0 PMC9490934

[14] G. Liu , X. Zeng , B. Wu , J. Zhao , and Y. Pan , “RNA‐Seq Analysis of Peripheral Blood Mononuclear Cells Reveals Unique Transcriptional Signatures Associated With Radiotherapy Response of Nasopharyngeal Carcinoma and Prognosis of Head and Neck Cancer,” Cancer Biology & Therapy 21, no. 2 (2020): 139–146. 31698994 10.1080/15384047.2019.1670521 PMC7012055

[15] Y. Wei , V. L. Z. Hui , Y. Chen , R. Han , X. Han , and Y. Guo , “YAP/TAZ: Molecular Pathway and Disease Therapy,” MedComm (2020) 4, no. 4 (2023): e340. 37576865 10.1002/mco2.340 PMC10412783

[16] Y. Qian , Z. Ma , Z. Xu , et al., “Structural Basis of Frizzled 4 in Recognition of Dishevelled 2 Unveils Mechanism of WNT Signaling Activation,” Nature Communications 15, no. 1 (2024): 7644. 10.1038/s41467-024-52174-z PMC11369211 39223191

[17] D. Chen , R. Xie , B. Shu , et al., “Wnt Signaling in Bone, Kidney, Intestine, and Adipose Tissue and Interorgan Interaction in Aging,” Annals of the New York Academy of Sciences 1442, no. 1 (2019): 48–60. 30101565 10.1111/nyas.13945 PMC6372353

[18] G. P. Solis , A. M. Luchtenborg , and V. L. Katanaev , “Wnt Secretion and Gradient Formation,” International Journal of Molecular Sciences 14, no. 3 (2013): 5130–5145. 23455472 10.3390/ijms14035130 PMC3634490

[19] A. Li , Y. Shen , Z. Li , et al., “Canonical Wnt Signaling Affects Calcium Homeostasis in Serum‐Treated AC16 Cells Through MLN‐Mediated SERCA2a Regulation,” Journal of Molecular Cell Biology 17 (2025): mjaf050. 10.1093/jmcb/mjaf050 PMC13093114 41348974

[20] V. P. Čokić , R. D. Smith , A. Biancotto , C. T. Noguchi , R. K. Puri , and A. N. Schechter , “Globin Gene Expression in Correlation With G Protein‐Related Genes During Erythroid Differentiation,” BMC Genomics 14 (2013): 116. 23425329 10.1186/1471-2164-14-116 PMC3602204

[21] X. Qiu , J. Lin , B. Liang , Y. Chen , G. Liu , and J. Zheng , “Identification of Hub Genes and MicroRNAs Associated With Idiopathic Pulmonary Arterial Hypertension by Integrated Bioinformatics Analyses,” Frontiers in Genetics 12 (2021): 667406. 33995494 10.3389/fgene.2021.636934 PMC8117102

[22] L. I. Cárdenas‐Navia , P. Cruz , J. C. Lin , S. A. Rosenberg , and Y. Samuels , “Novel Somatic Mutations in Heterotrimeric G Proteins in Melanoma,” Cancer Biology & Therapy 10, no. 1 (2010): 33–37. 20424519 10.4161/cbt.10.1.11949 PMC3040832

[23] M. Shao , F. Wu , J. Zhang , et al., “Screening of Potential Biomarkers for Distinguishing Between Latent and Active Tuberculosis in Children Using Bioinformatics Analysis,” Medicine (Baltimore) 100, no. 5 (2021): e23207. 33592820 10.1097/MD.0000000000023207 PMC7870233

[24] H. Wu , A. Scholten , A. Einwich , H. Mouritsen , and K. W. Koch , “Protein‐Protein Interaction of the Putative Magnetoreceptor Cryptochrome 4 Expressed in the Avian Retina,” Scientific Reports 10, no. 1 (2020): 7364. 32355203 10.1038/s41598-020-64429-y PMC7193638

[25] L. J. Huang , C. Liu , L. Chen , et al., “Evaluation of Pyroptosis‐Associated Genes in Endometrial Cancer Utilizing a 101‐Combination Machine Learning Framework and Multi‐Omics Data,” Frontiers in Medicine (Lausanne) 12 (2025): 1590405. 10.3389/fmed.2025.1590405 PMC12176823 40538398

[26] M. J. Parsons , T. Tammela , and L. E. Dow , “WNT as a Driver and Dependency in Cancer,” Cancer Discovery 11, no. 10 (2021): 2413–2429. 34518209 10.1158/2159-8290.CD-21-0190 PMC8487948

[27] P. Song , Z. Gao , Y. Bao , et al., “Wnt/β‐Catenin Signaling Pathway in Carcinogenesis and Cancer Therapy,” Journal of Hematology & Oncology 17, no. 1 (2024): 46. 38886806 10.1186/s13045-024-01563-4 PMC11184729

[28] Z. Rong , L. Zhang , Z. Li , et al., “SIK2 Maintains Breast Cancer Stemness by Phosphorylating LRP6 and Activating Wnt/β‐Catenin Signaling,” Oncogene 41, no. 16 (2022): 2390–2403. 35277657 10.1038/s41388-022-02259-0

[29] J. Wei , X. Zheng , W. Li , X. Li , and Z. Fu , “Sestrin2 Reduces Cancer Stemness via Wnt/β‐Catenin Signaling in Colorectal Cancer,” Cancer Cell International 22, no. 1 (2022): 75. 35148781 10.1186/s12935-022-02498-x PMC8840770

[30] Z. Miao , X. Zhao , and X. Liu , “Hypoxia Induced β‐Catenin Lactylation Promotes the Cell Proliferation and Stemness of Colorectal Cancer Through the Wnt Signaling Pathway,” Experimental Cell Research 422, no. 1 (2023): 113439. 36464122 10.1016/j.yexcr.2022.113439

[31] W. Zhang , X. Ruan , Y. Li , et al., “KDM1A Promotes Thyroid Cancer Progression and Maintains Stemness Through the Wnt/β‐Catenin Signaling Pathway,” Theranostics 12, no. 4 (2022): 1500–1517. 35198054 10.7150/thno.66142 PMC8825597

[32] H. Zhao , T. Ming , S. Tang , et al., “Wnt Signaling in Colorectal Cancer: Pathogenic Role and Therapeutic Target,” Molecular Cancer 21, no. 1 (2022): 144. 35836256 10.1186/s12943-022-01616-7 PMC9281132

