# Supplementary material for: Decoding the Oncogenic Role of GNG10 in Colorectal Cancer: A Non‐Canonical Wnt Pathway–Driven Mechanism
Source: J Cell Mol Med. 2026 Jun 7;30(11):e71170. doi: 10.1111/jcmm.71170 (PMC13243697; doi:10.1111/jcmm.71170)
Supplement: Supplementary file 1 — Figure S1: Multivariate Cox proportional hazards regression analysis of GNG10 and clinicopathological characteristics in colorectal cancer. Forest plots displaying the hazard ratios (HRs) and 95% confidence intervals (CIs) for overall survival based on two multivariate models. (A) Model A assesses the prognostic value of GNG10 after adjusting for age, sex, and overall AJCC stage. (B) Model B assesses the prognostic value of GNG10 after adjusting for age, sex, and individual tumour‐node‐metastasis (TNM) stages. Black squares represent the point estimate of the HR, and horizontal lines denote the 95% CIs. The vertical dashed line corresponds to an HR of 1.0 (no effect). Variables with p < 0.05 were considered statistically significant. The results indicate that while advanced age and late tumour stages (AJCC Stage III/IV, N2, and M1) serve as significant independent risk factors, GNG10 expression does not retain independent prognostic significance when adjusted for these established macroscopic pathological parameters. Figure S2: Basal endogenous expression of GNG10 across normal and colorectal cancer cell lines. Quantitative real‐time PCR (qRT‐PCR) analysis of baseline GNG10 mRNA expression levels in a normal human colon epithelial cell line (FHC) and five human colorectal cancer (CRC) cell lines (HT29, RKO, DLD‐1, HCT116, and CACO2). GAPDH was used as the internal control for normalization. Data are presented as the mean ± SD from three independent experiments. ***p < 0.001 versus the FHC group. [file JCMM-30-e71170-s001.docx]

**
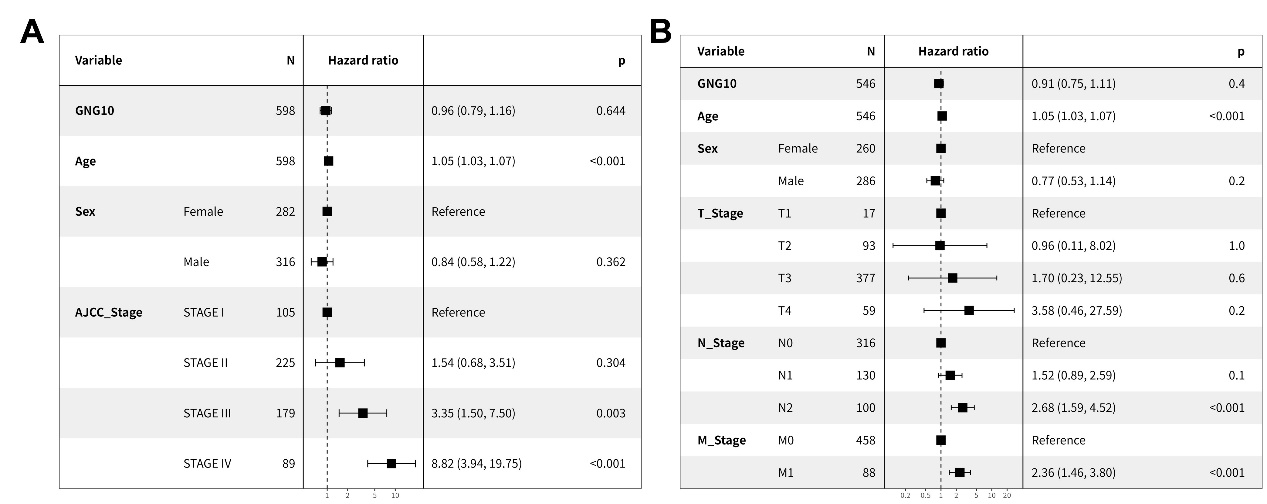
**

**Supplementary Figure S1. Multivariate Cox proportional hazards regression analysis of GNG10 and clinicopathological characteristics in colorectal cancer.**
Forest plots displaying the hazard ratios (HRs) and 95% confidence intervals (CIs) for overall survival based on two multivariate models. **(A)** Model A assesses the prognostic value of GNG10 after adjusting for age, sex, and overall AJCC stage. **(B)** Model B assesses the prognostic value of GNG10 after adjusting for age, sex, and individual tumor-node-metastasis (TNM) stages. Black squares represent the point estimate of the HR, and horizontal lines denote the 95% CIs. The vertical dashed line corresponds to an HR of 1.0 (no effect). Variables with *p* < 0.05 were considered statistically significant. The results indicate that while advanced age and late tumor stages (AJCC Stage III/IV, N2, and M1) serve as significant independent risk factors, GNG10 expression does not retain independent prognostic significance when adjusted for these established macroscopic pathological parameters.


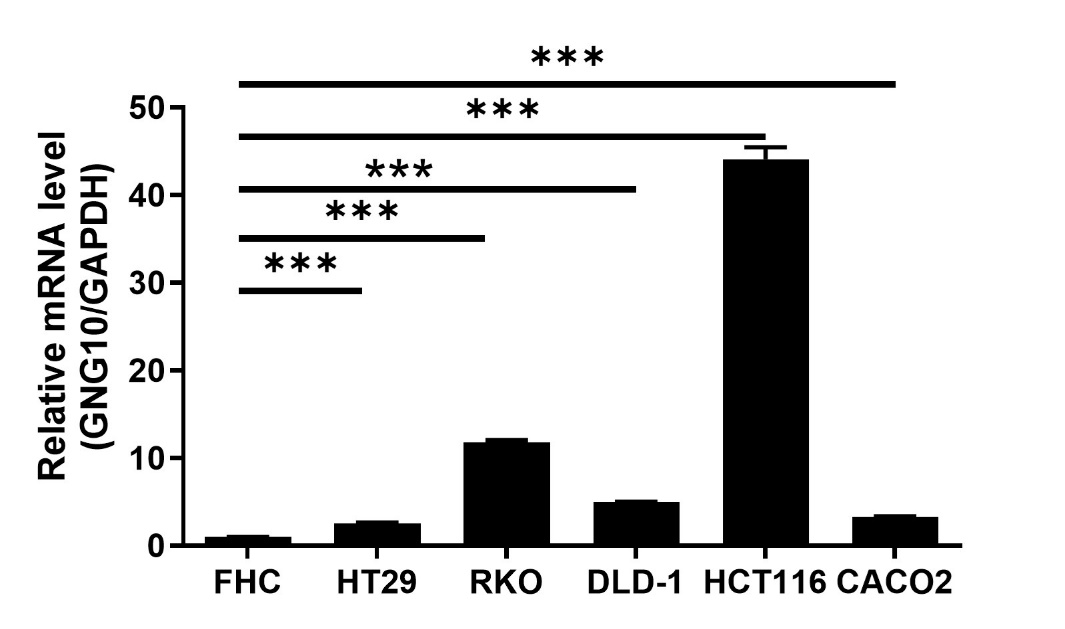


**Supplementary Figure S2. Basal endogenous expression of GNG10 across normal and colorectal cancer cell lines.**Quantitative real-time PCR (qRT-PCR) analysis of baseline GNG10 mRNA expression levels in a normal human colon epithelial cell line (FHC) and five human colorectal cancer (CRC) cell lines (HT29, RKO, DLD-1, HCT116, and CACO2). GAPDH was used as the internal control for normalization. Data are presented as the mean ± SD from three independent experiments. *** *p* < 0.001 vs. the FHC group.
